# Functional differentiation determines the molecular basis of the symbiotic lifestyle of *Ca*. Nanohaloarchaeota

**DOI:** 10.1186/s40168-022-01376-y

**Published:** 2022-10-14

**Authors:** Yuan-Guo Xie, Zhen-Hao Luo, Bao-Zhu Fang, Jian-Yu Jiao, Qi-Jun Xie, Xing-Ru Cao, Yan-Ni Qu, Yan-Lin Qi, Yang-Zhi Rao, Yu-Xian Li, Yong-Hong Liu, Andrew Li, Cale Seymour, Marike Palmer, Brian P. Hedlund, Wen-Jun Li, Zheng-Shuang Hua

**Affiliations:** 1 Chinese Academy of Sciences Key Laboratory of Urban Pollutant Conversion, Department of Environmental Science and Engineering, University of Science and Technology of China, Hefei, 230026 People’s Republic of China; 2 State Key Laboratory of Biocontrol, Guangdong Provincial Key Laboratory of Plant Resources and Southern Marine Science and Engineering Guangdong Laboratory (Zhuhai), School of Life Sciences, Sun Yat-Sen University, Guangzhou, 510275 People’s Republic of China; 3 State Key Laboratory of Desert and Oasis Ecology, Xinjiang Institute of Ecology and Geography, Chinese Academy of Sciences, Urumqi, 830011 People’s Republic of China; 4 School of Life Sciences, University of Nevada Las Vegas, Las Vegas, NV 89154 USA; 5 Nevada Institute of Personalized Medicine, University of Nevada Las Vegas, Las Vegas, NV 89154 USA

**Keywords:** *Cadidatus* Nanohaloarchaeota, Metabolism, Environmental adaptations, Functional differentiation

## Abstract

**Background:**

*Candidatus* Nanohaloarchaeota, an archaeal phylum within the DPANN superphylum, is characterized by limited metabolic capabilities and limited phylogenetic diversity and until recently has been considered to exclusively inhabit hypersaline environments due to an obligate association with *Halobacteria*. Aside from hypersaline environments, *Ca. *Nanohaloarchaeota can also have been discovered from deep-subsurface marine sediments.

**Results:**

Three metagenome-assembled genomes (MAGs) representing a new order within the *Ca. *Nanohaloarchaeota were reconstructed from a stratified salt crust and proposed to represent a novel order, *Nucleotidisoterales*. Genomic features reveal them to be anaerobes capable of catabolizing nucleotides by coupling nucleotide salvage pathways with lower glycolysis to yield free energy. Comparative genomics demonstrated that these and other *Ca.* Nanohaloarchaeota inhabiting saline habitats use a “salt-in” strategy to maintain osmotic pressure based on the high proportion of acidic amino acids. In contrast, previously described *Ca.* Nanohaloarchaeota MAGs from geothermal environments were enriched with basic amino acids to counter heat stress. Evolutionary history reconstruction revealed that functional differentiation of energy conservation strategies drove diversification within *Ca.* Nanohaloarchaeota, further leading to shifts in the catabolic strategy from nucleotide degradation within deeper lineages to polysaccharide degradation within shallow lineages.

**Conclusions:**

This study provides deeper insight into the ecological functions and evolution of the expanded phylum *Ca.* Nanohaloarchaeota and further advances our understanding on the functional and genetic associations between potential symbionts and hosts.

Video Abstract

**Supplementary Information:**

The online version contains supplementary material available at 10.1186/s40168-022-01376-y.

## Background

Archaea, as a vital component of Earth’s biodiversity, play a significant role in biogeochemical cycles and are a key partner in the evolutionary origin of eukaryotes [1, 2]. Most archaea are difficult to cultivate, limiting our understanding of their physiological and ecological properties [3]. Advances in metagenomics and single-cell genomics have significantly extended our understanding of the diversity and potential functions of uncultured archaea [4]. More than 20 novel uncultured phyla of archaea have been described in the past decade [5], as exemplified by the discovery of the Asgard and DPANN superphyla [6, 7]. A recent study divided the DPANN superphylum into seven *Candidatus* phyla, including Altiarchaeota, Iainarchaeota, Micrarchaeota, Undinarchaeota, Aenigmarchaeota, Nanohaloarchaeota, and Nanoarchaeota [8]. These lineages are united by very small cell sizes and small genome sizes with limited metabolic potential and are often considered to live in association with other microbes [9–11].

*Ca.* Nanohaloarchaeota represents a typical phylum in the DPANN superphylum that was first discovered in salt lakes and was originally identified as the sister branch of *Halobacteria* [12]. Using enrichment experiments and transmission electron microscopy, Hamm et al. demonstrated that some *Ca.* Nanohaloarchaeota cannot survive without *Halobacteria* partners [13]. A recent study revealed that the degradation of polysaccharides, including glycogen by some *Ca.* Nanohaloarchaeota, suggested that polysaccharide degradation might sustain a mutually beneficial interaction with its host *Halobacteria* [14]. *Ca.* Nanohaloarchaeota were originally thought to be exclusively derived from hypersaline habitats, and the observed taxa in these environments were limited to the order *Ca. *Nanosalinales [15–19]. However, a recent study discovered a novel *Ca.* Nanohaloarchaeota group in deep-subsurface marine sediment ecosystems [20], indicating that *Ca.* Nanohaloarchaeota may inhabit a wider range of habitats than previously understood and possibly harbor new functional niches that allow them to grow in different extreme environments. Thus, the diversity, metabolic potential, symbiotic lifestyles, and evolutionary adaptations to multiple extreme environments of the *Ca.* Nanohaloarchaeota are not completely understood.

By applying metagenomic sequencing, we reconstructed three *Ca.* Nanohaloarchaeota metagenome-assembled genomes (MAGs) from stratified salt crust samples. Phylogenomic analyses suggest that they represent a new order within *Ca.* Nanohaloarchaeota for which the name *Nucleotidisoterales* is proposed under the nascent SeqCode. Genomic analysis revealed that these MAGs lack genes for polysaccharide catabolism but instead encode complete nucleotide salvage pathways, suggesting that they might occupy a novel ecological niche and have an alternative strategy to interact with symbiotic partners. Ancestral character state reconstructions demonstrated that the last *Ca.* Nanohaloarchaeota common ancestor was unable to catabolize polysaccharides, and that shifts of energy conservation mechanisms led to the diversification of the *Ca.* Nanohaloarchaeota into multiple ecologically distinct lineages. This study represents a significant advancement in our understanding of the genomic diversity, ecology, and evolution of *Ca.* Nanohaloarchaeota.

## Results and discussion

### Overview of prokaryotic community structure

**Fig. 1:**
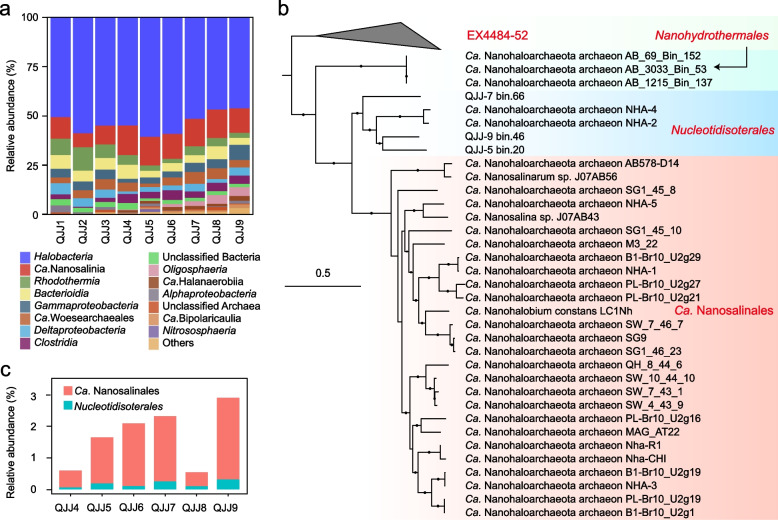
The reconstructed genomes of novel *Ca. *Nanohaloarchaeota. **a** Relative abundance of the major microbial groups in eight layers of a stratified salt crust and underlying water based on amplicon sequencing of the 16S rRNA gene. **b** Relative abundances of the three *Ca*. Nanohaloarchaeota MAGs recovered from the present study. **c** Phylogenetic placements of the novel *Ca*. Nanohaloarchaeota MAGs. The tree was constructed based on the concatenated alignment of 16 ribosomal proteins using IQ-TREE with the best model of LG + F + R9. Bootstrap values were based on 1000 replicates, and nodes with confidence > 70% are indicated as black circles

The Qi Jiao Jing (QJJ) Lake is a discharge playa lake located in Xinjiang Province of China, which has a salinity of more than 30% and is primarily composed of sodium, potassium, and chloride ions [21]. The high salinity forms a natural enrichment for halophiles with low species richness ranging from 143 to 181 ASVs (Fig. 1a). The microbial communities of the eight layers of the salt crust and one water column sample hosted similar microbial communities, but the organisms differed in their relative abundances (Fig. 1a). Archaea were more abundant than bacteria in all samples, with the middle layer (QJJ5) reaching the highest archaeal relative abundance (78.8%). *Halobacteria* and *Ca.* Nanohaloarchaeota predominated in all communities with relative abundances up to 60.7% and 15.1%, respectively. *Ca.* Woesearchaeales (1.7–6%) was the third most abundant group of archaea. Among bacteria, *Rhodothermia*, *Bacteroidia*, *Gammaproteobacteria*, *Deltaproteobacteria*, and *Clostridia* were the predominant groups, all of which are commonly found in saltern lakes [22]. Interestingly, the high abundance of *Oligosphaeria* in layers QJJ8 and QJJ9 suggests that they prefer the salt crust/water interface. Bacteria and archaea that were unclassified at the phylum level were also present in the salt crust community, indicating that there are still unknown microorganisms in high-salt environments.

### Identification of two novel orders of *Ca.* Nanohaloarchaeota

Three MAGs representing a novel lineage within *Ca.* Nanohaloarchaeota were reconstructed from the metagenomic datasets. The sizes of the assembled MAGs range from 0.62 to 0.75 Mbp with GC contents ranging from 43.8 to 52.5% (Table 1). They encode an average of 829 genes with an average gene length of 836 bp. The MAGs have high estimated completeness (87.5 to 95.8%; the occurrence frequencies of 48 single-copy marker genes used for genome completeness calculation are recorded in Additional file 1: Table S1) with up to 33 tRNAs and low estimated contamination (< 0.93%). Reads mapped to these MAGs are exclusively from the bottom six layers of the salt crust, with relatively low abundances, suggesting that they represent a rare group within the salt crust community (Fig. 1b). Given the significantly lower abundance of *Ca.* Nanohaloarchaeota MAGs compared with 16S rRNA-based data, we speculate that some *Ca.* Nanohaloarchaeota failed to assemble and/or bin into discrete MAGs, particularly for the *Ca. *Nanosalinales.

**Table 1:** Genomic features of *Ca*. Nanohaloarchaeota MAGs reconstructed from the Qi Jiao Jing Lake

Bin_ID	QJJ-5_bin.20	QJJ-7_bin.66	QJJ-9_bin.46
Genome size (bp)	701,335	753,270	625,082
No. of scaffolds	48	34	34
N50	23,342	30,141	22,555
GC	50.5	52.4	43.9
Coding density (%)	93.4	92.4	92.3
Predicted genes	846	889	753
No. of genes annotated by KO	375	373	339
No. of genes annotated by COG	194	217	196
No. of tRNAs	27	33	33
Completeness (%)^a^	87.5	95.8	89.6
Contamination^b^	0.07	0.93	0

^a^The genome completeness of each MAG was calculated as the occurrence frequency of 48 single-copy genes (see Additional file 1: Table S1)

^b^The contamination for each MAG was evaluated using CheckM (see “Materials and methods”)

To resolve the phylogenetic affiliation of the newly obtained MAGs, two different marker protein sets were used to construct phylogenomic trees using maximum-likelihood methods: (i) 16 ribosomal proteins (Fig. 1c) and (ii) 122 archaeal marker proteins (Additional file 2: Fig. S1). Both phylogenies are well supported and concordant with clustering patterns based on average amino acid identity (Additional file 2: Fig. S2). Based on monophyly with high bootstrap support and calculated relative evolutionary divergence (RED) values (0.57 ± 0.004; Additional file 1: Table S2), these MAGs, along with two unclassified MAGs derived previously from hypersaline environments, could be assigned into a novel order within the previously defined class *Ca. *Nanosalinia. Three additional publicly available MAGs obtained from the subsurface within a deep-sea hydrothermal vent area can also be classified into this class but represent a third order, herein called *Nanohydrothermales*. A system of nomenclature was developed under the rules of the SeqCode, with the orders *Nucleotidisoterales* and *Nanohydrothermales* proposed to encompass MAGs reconstructed from hypersaline environments and deep-sea hydrothermal vents, respectively. Under the SeqCode, the nomenclatural types for the orders are the genera *Nucleotidivindex* and *Nanohydrothermus*. In total, four families encompassing four monospecific genera are proposed. All proposed taxa are supported by phylogenetic concordance and delineated based on RED values and the average nucleotide identity cutoff of < 95% at the species level (Additional file 3).

### Nucleotidisoterales may be symbionts of *Halobacteria*

**Fig. 2:**
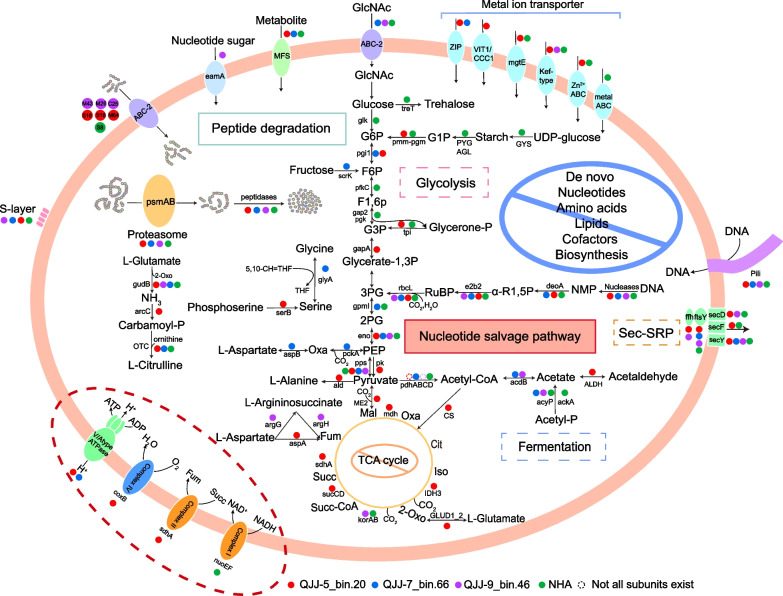
Overview of metabolic potentials in three new *Ca*. Nanohaloarchaeota MAGs. Genes related to glycolysis, AMP metabolism, TCA cycle, pyruvate metabolism, oxidative phosphorylation, protein degradation, membrane transporters, and pili are shown. Red solid circles represent genes present in QJJ-5_bin.20. Blue solid circles represent genes present in QJJ-7_bin.66. Purple solid circles represent genes present in QJJ-9_bin.46. Green solid circles represent genes present in at least one of the two NHA MAGs. Hollow circles represent structures where not all protein subunits are present in the genome. Abbreviations: G1P, glucose 1-phosphate; G6P, glucose 6-phosphate; F6P, fructose 6-phosphate; F1,6P2, fructose 1,6-bisphosphate; G3P, glyceraldehyde-3p; 3PG, glycerate-3-phosphate; 2PG, glycerate-2-phosphate; PEP, phosphoenolpyruvate; Oxa, oxaloacetate; Cit, citrate; Iso, isocitrate; 2-Oxo, 2-oxo-glutarate; Succ-CoA, succinate-CoA; Succ, succinate; Fum, fumarate; Mal, malate; NMP, nucleoside 5′-monophosphate; R1,5P, ribose-1,5-bisphosphate; RuBP, ribulose-1,5-disphosphate; MFS, major facilitator superfamily permease; 5,10-CH = THF, 5,10-methylenetetrahydrofolate; Carbamoyl-P, carbamoyl phosphate; phosphoserine, 3-phospho-L-serine

Analysis of the metabolic potential of *Nucleotidisoterales* genomes showed that they have genes for DNA replication, transcription, and translation but lack biosynthetic capacity to synthesize nucleotides, amino acids, lipids, and cofactors (Fig. 2). These gaps in essential biosynthetic pathways, particularly cell membrane biosynthesis, imply that they likely have a symbiotic lifestyle [9]. Also, in support of this hypothesis are the extremely small genome sizes of all five *Nucleotidisoterales* genomes, 0.62 to 0.75 Mbp, which are much smaller than any known free-living prokaryotes, and the general lack of evidence for a free-living lifestyle in any other member of the DPANN superphylum. Based on the abundance and diversity of cohabiting *Halobacteria* and known examples of symbioses between *Ca.* Nanohaloarchaeota and *Halobacteria*, members of the *Nucleotidisoterales* may be obligate symbionts of *Halobacteria*; however, this hypothesis would need to be resolved by more incisive experiments.

### Metabolic potential of novel MAGs

The incomplete TCA cycle and the absence of most electron transport components reveal most members of this lineage are fermentative and anaerobic, in agreement with previous analyses of other *Ca.* Nanohaloarchaeota genomes and all other members of the DPANN superphylum [9, 11]. Notably, QJJ-5_bin.20 encodes cytochrome c oxidase, *coxB*, though other subunits of complex IV including *coxACD* were absent. It has been reported that the presence of cytochrome oxidase encoded by some DPANN genomes may be employed to adapt to oxic or microoxic environments [9, 10]. Genes involved in the oxidative and non-oxidative pentose phosphate pathways and upper glycolytic pathway are almost completely missing (Fig. 2, detailed gene copies are recorded in Additional file 1: Table S3). However, all MAGs encode complete nucleotide salvage pathways, including AMP phosphorylase (*deoA*), ribose 1,5-bisphosphate isomerase (*e2b2*), and form-III type of ribulose 1,5-bisphosphate carboxylase (*rbcL*), suggesting that they could degrade adenosine monophosphate (AMP) or nucleoside 5′-monophosphate (NMP) into 3-phosphoglycerate, which could feed into the lower glycolytic pathway with ATP released [23]. Notably, none of the previously described *Ca.* Nanohaloarchaeota genomes has been reported to contain this pathway, despite its common presence in other DPANN archaea [9, 24].

**Fig. 3:**
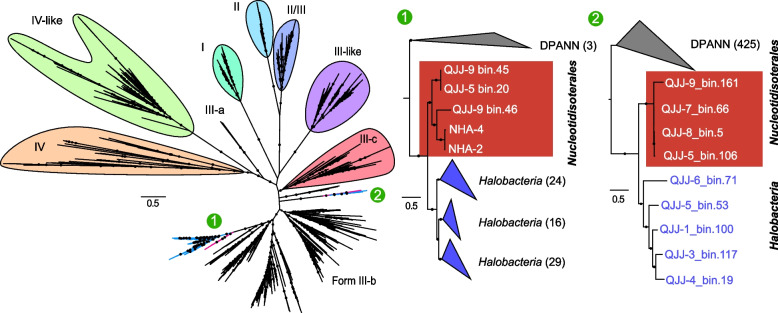
Maximum-likelihood phylogenetic tree of RbcL proteins using IQ-TREE with the best model of LG + F + R10. **a** Overview of RbcL proteins phylogeny with groups indicated. **b** and **c** Detailed phylogeny of two different clades of RbcL proteins found in *Nucleotidisoterales*; red highlights represent the *Nucleotidisoterales* MAGs. QJJ-9_bin45, QJJ-9_bin.161, QJJ-8_bin.5, and QJJ-5_bin.106 represent medium- or low-quality MAGs of *Nucleotidisoterales* reconstructed from the same metagenomic datasets and thus not described in the main text. Bootstrap values were based on 1000 replicates, and nodes with percentages > 70% are indicated as black circles

Phylogenetic analysis showed that the RbcL proteins of *Nucleotidisoterales* formed two groups within the form III-B lineage (Fig. 3), both of which are mainly composed of homologs from other DPANN archaea. Given the frequent horizontal gene transfers (HGTs) among DPANN archaea and very limited distribution of RbcL in *Ca*. Nanohaloarchaeota [24], we infer that the common ancestor of the RbcL homologs within *Nucleotidisoterales* might be endowed by other DPANN. Additionally, RbcL homologs annotated in several *Halobacteria* genomes were also divided into two groups, and both are located as sister lineages of RbcL proteins in the *Nucleotidisoterales*. Based on the wide presence of RbcL proteins in DPANN archaea and the close evolutionary relationship between homologs from *Nucleotidisoterales* and *Halobacteria*, we speculate that lineage III-B evolved within DPANN and passed to *Halobacteria* horizontally with *Ca.* Nanohaloarchaeota acting as potential donors. Rampant nucleotide scavenging is well-known in *Halobacteria* [25]. This also exemplifies that the bidirectional genetic exchange between *Ca.* Nanohaloarchaeota and co-existing, and possibly symbiotic, *Halobacteria* is probable.

*Nucleotidisoterales* may obtain DNA through pili (see below) or by direct transport from other organisms and then degrade it by use of nucleases [26]. We hypothesize that *Nucleotidisoterales* and *Halobacteria* may collaborate to degrade DNA, and the resulting oligonucleotides or nucleotides can be recycled by *Nucleotidisoterales* via the nucleotide salvage pathway. The glycerate-3P produced could flow into the lower glycolytic pathway, leading to pyruvate. This is feasible due to the possession of all genes encoding the lower glycolytic pathway, including a 2,3-bisphosphoglycerate-independent phosphoglycerate mutase (*gpmI*), enolase (*eno*), and phosphoenolpyruvate synthase (*pps*). Then, acetate could be transported into acetotrophic *Halobacteria*, which may be potential hosts [27]. *Nucleotidisoterales* lack the archaeal pyruvate reductase (*porAB*) for the conversion of pyruvate to acetyl-CoA. Instead, the pyruvate dehydrogenase complex (*pdhABCD*), which is commonly detected in DPANN [9], might alternatively be employed to perform the same function. The presence of acetate-CoA ligase (*acdB*) suggests the capability to catalyze the reversible conversion of acetyl-CoA and ADP to acetate and ATP, which was shown to be operational in *Ca.* Nanohalobium constans LC1Nh [14]. Alternatively, aldehyde dehydrogenase might be adopted to produce acetate and NADH by oxidizing acetaldehyde. Thus, we suggest a potential syntrophic relationship between the putative host/symbiont partners. Under this hypothesis, symbionts in the order *Nucleotidisoterales* may obtain diverse nutrients from hosts, and in turn, they may provide some small molecules, such as acetate, to acetotrophic *Halobacteria* hosts [27, 28]. Collectively, we hypothesize that *Nucleotidisoterales* organisms are obligate symbionts with the capability for nucleotide fermentation. Aside from the known co-metabolism of carbohydrates by *Ca. *Nanosalinales and *Halobacteria* partners, co-degradation of extracellular DNA by *Nucleotidisoterales* via the nucleotide salvage pathway, coupled with the lower glycolytic pathway and acetogenesis, might represent another strategy to support mutualistic symbiosis.

### Metabolism of polysaccharides and peptides

A previous study demonstrated that the pure culture *Halomicrobium* sp. LC1Hm is unable to grow with glycogen as the sole carbon source. Instead, the co-cultured symbiont *Ca.* Nanohalobium constans LC1Nh can assist the degradation of glycogen, and the released glucose supports the growth of *Halomicrobium* sp. LC1Hm [14]. This interaction serves as an example of how microbial consortia often have a broader metabolic capacity than pure cultures, allowing the survival of partners in the relationship despite resource fluctuations or environmental disturbances. Unlike the co-culture of *Ca.* Nanohalobium constans LC1Nh and *Halomicrobium* sp. LC1Hm where the coexistence is maintained by the utilization of different polysaccharides, none of our *Nucleotidisoterales* MAGs encodes genes for glycoside hydrolases (Additional file 1: Table S4), further suggesting that a novel strategy might be employed to sustain a symbiotic relationship.

Instead of polysaccharides, *Nucleotidisoterales* may be able to utilize proteins or peptides by use of a variety of protein-degrading enzymes for catabolic and/or anabolic purposes. Specifically, two subunits of the proteasome (*psmAB*), as well as several molecular chaperones (Fig. 2), were present, indicating that they have the ability to degrade damaged or misfolded proteins into oligopeptides. Different families of peptidases were detected, which could participate in the processing and transport of oligopeptides or protein turnover (Additional file 1: Table S5), such as serine peptidase (e.g., S16 and S26), metallopeptidases (e.g., M26, M43, and M103), aspartic peptidase (e.g., A26), and other peptidases (e.g., U32, T01, and N10). Moreover, six peptidases (S16, S18, M26, M43, M64, and C25) carry signal peptides (Additional file 1: Table S6), suggesting that they could degrade peptides extracellularly into oligopeptides or amino acids that could be transported by the MSF transporter or ABC-2 transporter [14] (Fig. 2). Several pathways for the catabolic use of amino acids and interconversion of amino acids have been identified, implying that proteolysis is an important way of life for *Nucleotidisoterales*. For example, not only may glutamate be deaminated to generate ammonia but also it may be used as a compatible solute to regulate intracellular osmotic pressure [29]. Aspartate could potentially be converted into oxaloacetate and then used to produce acetate. This indicates that oligopeptides and amino acids are likely to be important substrates for the growth of *Nucleotidisoterales*.

### Cell-surface structures

A pioneering study suggested that a prominent feature of *Ca.* Nanohaloarchaeota is large genes that encode the so-called SPEARE proteins, which usually contain several domains thought to be involved in the interaction between symbionts and hosts [10]. However, no open reading frames larger than 9000 nucleotides were annotated in any of the *Nucleotidisoterales* MAGs, yet several small genes encoding SPEARE-like proteins were identified (Additional file 1: Table S6). Therefore, *Nucleotidisoterales* may use different strategies to promote cell–cell interactions. Studies have shown that pili and archaella, as well as certain surface proteins, likely contribute to cell surface attachment and interaction between symbionts and hosts [30, 31]. Unlike other *Ca.* Nanohaloarchaeota, none of MAGs in the present study encodes archaella. All *Nucleotidisoterales* MAGs have at least two type 4 tight adherence (Tad) pilus-encoding gene clusters putatively involved in pilus formation with one cluster being identical in architecture and gene organization to that found in all known *Ca*. Undinarchaeota (Additional file 2: Fig. S3) [26]. Specifically, VirB11 family ATPases (TadA) are possibly used to energize the assembly and disassembly of pili by hydrolyzing nucleotide triphosphates [32] TadB and TadC could potentially provide a platform for pilus assembly [33] and type 4 prepilin peptidases (TadV) used to modify prepilins. However, no pilins were annotated in these genomes, possibly due to the small size and poor conservation of pilin genes. By comparison, *Ca.* Undinarchaeota, which also lack annotated pilin genes, have been confirmed to synthesize pili to promote cell–cell interactions [26]. The other two types of gene clusters in the new MAGs contain two copies of *kaiC* but lack prepilin peptidase*.* The *kaiC* genes are possibly involved into the regulatory or modulation of type 4 pilin [34]. Studies have also shown that several DPANN archaea may interact with hosts and import DNA into cells via pili [26, 31]. Consequently, we hypothesize that the pili of *Nucleotidisoterales* are not only used for communication with the host but also for nutrient transport, similar to all known *Ca*. Undinarchaeota [26]. In addition, signal-peptide-containing proteins, including LamG domain-containing proteins, glycosyltransferases, S-layer family proteins, and several hypothetical proteins, were identified (Additional file 1: Table S7), which could be transported outside by the Sec-SRP secretion system to promote cell–cell interactions [9]. Metal ion transporters were detected, albeit at low abundance, except for QJJ-5_bin.20, which contains five different metal transporters (Fig. 2). This includes several magnesium transporters, which have been experimentally confirmed to support the cell growth of *Ca.* Nanohaloarchaeota due to their reliance on high cytoplasmic magnesium [14].

### Environmental adaptations

**Fig. 4:**
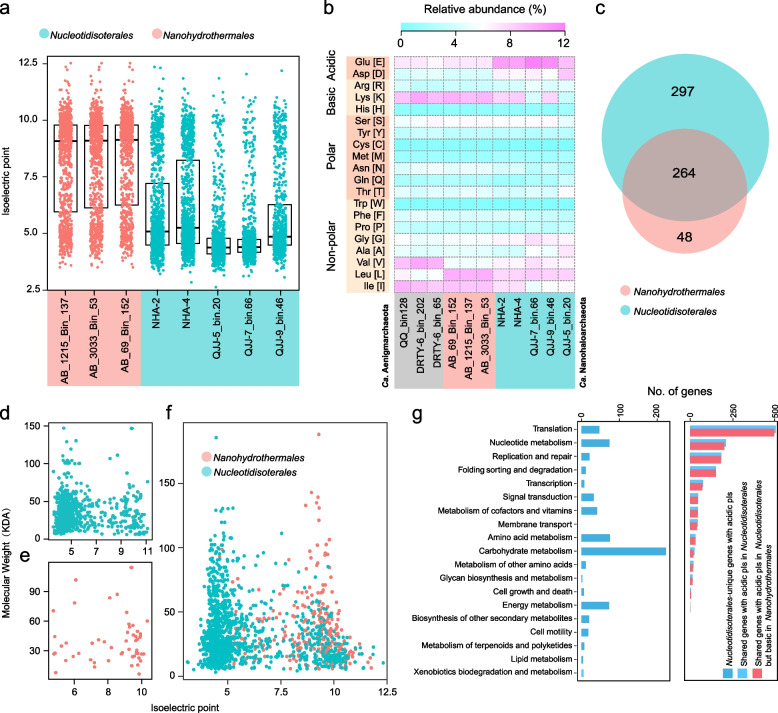
Genomic differences between *Nucleotidisoterales* and *Nanohydrothermales*. Five MAGs from *Nucleotidisoterales* and three MAGs from *Nanohydrothermales* are taken into consideration for comparative genomics. **a** The isoelectric points (pIs) of the proteins of MAGs from the two orders (see “Materials and methods” for detailed calculation of pI). **b** Amino acid usage of MAGs, as well as thermophilic *Ca*. Aenigmarchaeota. **c** The Venn diagram indicates differences between the two orders at the KO level. **d** Isoelectric points of *Nucleotidisoterales*-unique proteins. **e** Isoelectric points of *Nanohydrothermales*-unique proteins. **f** Isoelectric points of proteins shared between *Nucleotidisoterales* and *Nanohydrothermales*. **g** Functional distribution of acidic amino acids at the KEGG category level. Blue bars represent unique genes of *Nucleotidisoterales*. Light blue bars represent shared genes of *Nucleotidisoterales*. Red bars represent shared genes of *Nanohydrothermales*

To sustain isoosmosis with the surrounding environment, different strategies might be used to maintain proper intracellular osmotic pressure. Salt-in is a key strategy used by *Halobacteria*, which leads to the proteomic enrichment of acidic and hydrophilic amino acids [35, 36]. Calculations of the average isoelectric points (pIs) of all protein-encoding genes yielded very low median pI values (average 4.5), confirming the salt-in strategy adopted by *Nucleotidisoterales*, similar to *Ca.* Nanosalinales [14] (Fig. 4a). In contrast, *Nanohydrothermales* MAGs possess more basic proteomes with high median pI values (average 8.94) [37]. Detailed investigation of amino acid usages revealed substantial differences between *Nucleotidisoterales* and *Nanohydrothermales* (Fig. 4b). The former possesses a high excess of surficial acidic amino acids (Glu, Asp), which are able to enhance hydration to keep the proteins in solution [38]. Moreover, these negatively charged amino acids bind to specific cations (e.g., Na^+^ and K^+^) to maintain structural stability and enzyme activity [39, 40]. *Nanohydrothermales* MAGs have amino acid compositions similar to those of thermophilic *Ca*. Aenigmarchaeota (Fig. 4b). The charged amino acid lysine is enriched, but uncharged polar amino acids are in low relative abundances (Ser, Thr, Gln, and Asn) [41]; lysine methylation could enhance protein stability under high-temperature conditions [42].

Comparative genomics based on KEGG annotation revealed remarkable differences between the two orders (Fig. 4c). More genes could be assigned to KOs in *Nucleotidisoterales* compared with *Nanohydrothermales*, and the former group harbors more unique genes than the latter. In contrast, shared genes comprise 84.6% of *Nanohydrothermales* genomes. Both unique and shared genes within respective groups exhibited a similar pattern of pI values (Fig. 4 d–f). Interestingly, regardless of whether the genes are unique or not, *Nucleotidisoterales* MAGs are enriched in acidic amino acids with relatively low molecular weights. Unique genes with low pI in *Nucleotidisoterales* MAGs are involved in carbohydrate, amino acid, nucleotide, and energy metabolisms. In contrast, shared genes in *Nucleotidisoterales* exhibiting low pI values were enriched in translation, nucleotide metabolism, and replication and repair (Fig. 4g). Interestingly, most of these shared genes with low pI values in *Nucleotidisoterales* are basic with pI values > 7 in *Nanohydrothermales*, demonstrating divergent evolution of these genes to adapt to their distinct habitats. Collectively, the enriched genes and amino acid patterns appear primarily determined by the distinct physicochemical environments harboring these two orders.

### Evolution of carbohydrate metabolism in *Ca. *Nanohaloarchaeota

**Fig. 5:**
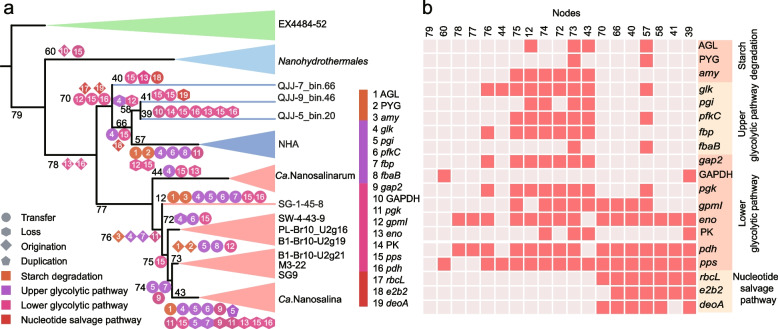
Evolutionary history reconstruction regarding carbohydrate metabolism and nucleotide salvage pathway in *Ca*. Nanohaloarchaeota. **a** The inferred gain and loss events related to carbohydrate metabolism and nucleotide salvage pathway in *Ca.* Nanohaloarchaeota. **b** The presence and absence of genes in each *Ca.* Nanohaloarchaeota MAG. Originations indicate either de novo gene birth events or inter-phylum HGTs. NHA represents *Ca*. Nanohaloarchaeota archaeon NHA-2 and *Ca*. Nanohaloarchaeota archaeon NHA-4

A reassessment of the evolutionary history of *Ca.* Nanohaloarchaeota is necessary due to the discovery of *Nucleotidisoterales* described here and the recently discovered *Nanohydrothermales* from deep-sea hydrothermal vents. Including all *Ca*. Nanohaloarchaeota MAGs available in public databases, the reconstructed phylogeny reveals the deep-branching position of the *Nanohydrothermales* and a sister group encompassing *Nucleotidisoterales* and *Ca. *Nanosalinales (Fig. 5). Due to the demonstrated importance of polysaccharide utilization for the establishment of symbiotic relationships in some members of the *Ca.* Nanosalinales, genes involved in carbohydrate metabolism along with nucleotide metabolism were considered for evolutionary history inference. The reconstructed evolutionary history based on carbohydrate-related metabolisms revealed different evolutionary trajectories among different orders within the *Ca. *Nanohaloarchaeota that inhabit thermal and high-salt habitats. Specifically, the metabolic characteristics of *Nanohydrothermales* are distinct from other members of the phylum because of lacking pathways for sugar catabolism. The lower glycolytic pathway appears to be fundamental and widely distributed across both orders inhabiting high-salt environments. Specifically, the *eno* and *pdhABCD* genes were likely already present at the ancestral node of *Nucleotidisoterales* and *Ca*. Nanosalinales, and other genes, including *gpmI* and *pps*, are also found in the early stages of *Nucleotidisoterales* lineages (Fig. 5). Functional differentiation occurred after the two lineages diversified. In particular, genes encoding proteins involved in polysaccharide metabolism, such as glycogen debranching enzyme (AGL) and alpha amylase (*amy*), appear to be acquired features in *Ca*. Nanosalinales, which was critical for the evolution of symbiotic polysaccharide metabolism between the hosts and symbionts as reported [14]. Phylogenetic analysis suggests that *Ca*. Nanosalinales may have acquired these genes from other DPANN archaea via HGT (Additional file 2: Figs. S4 and S5). Meanwhile, *Ca*. Nanosalinales evolved the upper glycolysis pathway mostly driven by HGT, facilitating the degradation of polysaccharides, whose products then flow into a complete glycolysis pathway to conserve energy. Genes associated with carbohydrate metabolism were mostly acquired horizontally, suggesting the inability of the last *Ca*. Nanohaloarchaeota common ancestor to metabolize saccharides to obtain free energy. However, none of our *Nucleotidisoterales* MAGs harbors genes for the complete polysaccharide metabolism or the upper glycolytic pathway. In contrast, the nucleotide salvage pathway, conferring microbes with the ability to harvest energy by degrading nucleotides, is exclusively detected in *Nucleotidisoterales* within *Ca*. Nanohaloarchaeota. The three key genes (*rbcL*, *deoA*, and *e2b2*) involved in this pathway seem to be inherited vertically from their common ancestor, with few HGT events (Fig. 5). However, we cannot rule out the possibility that the common ancestor of *Nucleotidisoterales* may acquire these genes via inter-phylum HGT. This is probable which could be exemplified by the aforementioned evolution of *rbcL* gene. *Ca. *Nanohaloarchaeota thus differentiated into separate branches based on the different strategies for energy conservation. *Ca*. Nanosalinales conserve energy by degrading starch coupled with complete glycolysis and fermentation, whereas *Nucleotidisoterales* conserve energy via nucleotide salvage coupled with lower glycolysis. The nucleotide salvage pathway is much more energy efficient than the upper glycolysis pathway because it can produce two more ATPs per reaction. However, a study found that the activity of AMP phosphorylase and ribose-1,5-bisphosphate isomerase increased only at higher substrate concentrations, preventing the excessive degradation of intracellular nucleotides [43]. Additionally, glycolysis is capable of rapidly supplying energy [44], and likely represents a more effective manner to support cell growth. This could manifest in the much higher abundance of *Ca*. Nanosalinales than *Nucleotidisoterales* in the community studied here.

## Conclusion

We provide a comprehensive analysis of the potential metabolism, ecology, and evolution of a novel order of *Ca. *Nanohaloarchaeota, *Nucleotidisoterales*. Unlike other *Ca. *Nanohaloarchaeota, *Nucleotidisoterales* are unable to degrade polysaccharides. Instead, genomic analysis suggests they can recycle and degrade nucleotides and proteins for anabolism and energy conservation, suggesting that they occupy different ecological niches. This is the first description of the nucleotide salvage pathway in *Ca.* Nanohaloarchaeota genomes, and phylogenetic analysis revealed that *Nucleotidisoterales* are possible donors of *rbcL* genes to* Halobacteria*. Comparative genomic analysis revealed remarkable differences between *Nucleotidisoterales* and thermophilic *Nanohydrothermales*, including amino acid usage, suggesting different physicochemical environments selected for distinct proteomes to thrive in different extreme environments. Evolutionary history analysis suggested that the last *Ca.* Nanohaloarchaeota common ancestor was unable to metabolize polysaccharides for energy conservation, and that later functional differentiation with respect to energy harvesting led to the diversification of *Ca.* Nanohaloarchaeota. Overall, the findings provide deeper insights into the understanding of the metabolic functions and the evolutionary history of the important but poorly studied phylum *Ca.* Nanohaloarchaeota.

## Materials and methods

### Sampling, DNA extraction, 16S rRNA amplicon, and metagenomic sequencing

A stratified salt crust ~ 14 cm thick was sampled from the surface of the Qi Jiao Jing (QJJ) Lake in Xinjiang Province of China (91.5881°E, 43.3806°N), in September 2019. The crust was characterized by distinct colored layers due to photosynthetic pigments and different mineral phases, and the bottom layer was at the interface with the underlying water (Additional file 2: Fig. S6). The salt crust was dissected into eight distinctive layers based on differences in color (QJJ1-8), and one sample from the underlying salt water was also collected (QJJ9). All nine samples were placed into 15 ml sterile tubes and stored in liquid nitrogen during shipment to the lab. DNA was extracted within 48 h as described previously [45]. The standard primer 515F-806R for the V4 region of the 16S rRNA gene was used for DNA amplification [46, 47]. High-throughput sequencing was performed on an Illumina MiSeq 2500 platform to generate 250 bp paired-end reads. Separately, a library with an insert size of ~ 400 bp was constructed from the total genomic DNA and was sequenced using the Illumina HiSeq 2500 instrument, generating ~ 36 Gbp (2 × 150 bp) raw data for each sample.

### Analyses of 16S rRNA gene amplicons

The adapters and low-quality reads were removed by cutadapt v1.18 [48]. Clean data were processed according to the recommended tutorial in the QIIME2 program (2020.7) [49]. In brief, sequences were merged into amplicon sequence variants (ASVs) after demultiplexing, joining, filtering, and denoising. ASVs were taxonomically identified using the QIIME2 classifier by searching against the SILVA v132 database [50].

### Processing metagenomic reads, assembly, and binning

All metagenomic raw reads were filtered by Sickle v1.33 (https://github.com/najoshi/sickle) with the parameters “-q 15 -l 50.” High-quality reads were assembled using SPAdes v3.12 [51] with the parameters “-k 21, 33, 55, 77, 99 -meta.” Genome binning was conducted on scaffolds with lengths ≥ 2500 bp using MetaBAT2 with default parameters [52]. The taxonomy of MAGs was obtained based on the GTDB database using GTDB-Tk v1.7.0 [53, 54]. Three MAGs belonging to *Ca. *Nanohaloarchaeota were retained for further analysis. The MAGs were evaluated for completeness by calculating the proportion of detected markers among 48 single-copy genes [55]. Contamination was assessed using CheckM v1.1.3 [56]. To optimize MAG quality, clean reads for each MAG were recruited using BBMap v38.92 (http://sourceforge.net/projects/bbmap/) with the parameters “minid = 0.97, local = t.” Then, MAGs were reassembled by SPAdes v3.12 based on the mapped reads with the following parameters: “–careful -k 21, 33, 55, 77, 99, 127.” To improve the accuracy of genome bins, all bins were manually examined to remove contamination. Specifically, scaffolds were treated as contamination and were discarded if they contained duplicate markers that were phylogenetically discordant with other *Ca*. Nanohaloarchaeota, and their read depths were discordant with other scaffolds in the same bin. The relative abundance of each MAG in each sample was calculated by calculating the proportion of reads mapped to each MAG against all preliminary genomes generated via MetaBAT2.

### Functional annotation of *Ca. *Nanohaloarchaeota MAGs

All the available MAGs belonging to *Ca. *Nanohaloarchaeota were downloaded from NCBI, IMG, and other sources (Additional file 1: Table S2) [19]. MAGs with completeness < 50% and contamination > 10% were discarded. Pairwise average amino acid identity among MAGs was calculated as the mean identity of reciprocal best BLAST hits (*E*-value < 1e-5). rRNAs and tRNAs were identified using RNAmmer v1.2 and tRNAscan-SE v2.0.2, respectively [57, 58]. Putative protein-coding sequences were predicted using Prodigal v2.6.3 with the “ -p single” parameter [59]. Subsequently, predicted genes were annotated against KEGG, NCBI-nr, and eggNOG databases using DIAMOND (*E*-values < 1e-5) [60]. Carbohydrate-active enzymes were annotated using the carbohydrate-active enzymes (CAZy) database [61]. Peptidases were identified by BLAST searching against the MEROPS database [62]. SignalP-4.1 was used to predict signal peptides and the localization of the enzymes [63]. The isoelectric point of the proteins was calculated using IPC v1.0 [64].

### Phylogenetic analyses

Two different marker gene sets were used to analyze relationships between members of the *Ca. *Nanohaloarchaeota. First, sixteen ribosomal protein sequences (L2, L3, L4, L5, L6, L14, L15, L16, L18, L22, L24, S3, S8, S10, S17, and S19) were selected to reconstruct phylogenomic relationships [65]. These sequences were identified by AMPHORA2 [66] and aligned using MUSCLE v3.8.31 by iterating 100 times [67]. Poorly aligned regions were eliminated using TrimAl v1.4 with the parameters “-gt 0.95 -cons 50” [68]. Then, multiple alignments were concatenated using a Perl script (https://github.com/nylander/catfasta2phyml). Second, a multiple sequence alignment of 122 archaea-specific conserved marker genes generated by GTDB-Tk v1.7.0 [53] was used for phylogenetic analysis. To build a phylogenetic tree of the ribulose 1,5-bisphosphate carboxylase (RbcL), reference protein sequences were obtained from a previous study and the NCBI-nr database [24]. RbcL proteins belonging to *Halobacteria* were identified from metagenomic data in the present study and were integrated to assess the evolution of this gene. All RbcL protein sequences within *Halobacteria* were clustered using CD-HIT v4.8.1 with the parameters “-c 0.95 -n 10 -G 0 -aS 0.9 -g 1 -d 0 -T 20” [69]. IQ-TREE v1.6.12 was applied to reconstruct maximal-likelihood phylogenetic trees with the following parameters “-alrt 1000 -bb 1000” [70]. Phylogenetic trees for the glycogen debranching enzyme and alpha amylase were generated similarly. All the tree files were uploaded to iTOL for visualization and annotation [71].

### Evolutionary analysis

Protein families were obtained by applying the MCL algorithm (v14–137) to all *Ca*. Nanohaloarchaeota genomes [72]. Individual phylogenetic analyses for each protein family were constructed using the methods described above. To address the evolutionary history of *Ca*. Nanohaloarchaeota, gene gain and loss events were inferred by reconciling the topology difference between species tree and protein trees using ALE v1.0 [73]. ALEobserve was used to calculate the conditional clade probabilities from bootstrap samples, and 100 reconciliations with the species tree were sampled by ALEml_undated [74]. We used auxiliary scripts (https://github.com/Tancata/phylo/tree/master/ALE) to parse ALE outputs. A threshold of 0.3 was applied to the raw reconciliation frequencies of ALE output to judge whether an evolutionary event occurred or not [75]. If the gene copy parameter was greater than 0.3, the gene was considered to be present. Since noise from alignments and tree reconstructions can reduce the signal of some true events, this threshold is necessary [75].

## Supplementary Information

**Additional file 1:** **Supplementary Table S1-S7.** **Table S1.** The occurrence frequency of 48 single-copy genes and estimated genomic completeness of all *Ca.* Nanohaloarchaeota genomes. **Table S2.** Basic genomic features of publicly available Nanohaloarchaeota genome. **Table S3.** KEGG-based functional annotation of five *Nucleotidisoterales* MAGs including three from Qijiaojing lake and two from public database. **Table S4.** Gene counts of carbohydrate metabolism related genes identified by comparing to CAZy database. **Table S5.** Detected peptidases in *Nucleotidisoterales* MAGs. **Table S6.** Psiblast results of SPEARE proteins in *Ca.* Nanohaloarchaeota genomes. **Table S7.** Identification of signal peptides in genes predicted from *Nucleotidisoterales* MAGs.

**Additional file 2:** **Supplementary Fig. S1-S6.** **Supplementary Fig. S1.** | Phylogenetic placement of *Ca*. Nanohaloarchaeota MAGs based on 122 concatenated archaeal protein markers. **Supplementary Fig. S2.** | Pairwise comparisons of average amino acid identities among all *Ca.* Nanohaloarchaeota genomes. **Supplementary Fig. S3.** | The gene clusters related to the pili biosynthesis. **Supplementary Fig. S4.** | Maximum likelihood-based phylogenetic tree of alpha amylase encoded by *amy* using IQ-TREE with the best model of LG+R5. **Supplementary Fig. S5.** | Maximum likelihood-based phylogenetic tree of glycogen debranching enzyme encoded by AGL using IQ-TREE with the best model of LG+F+R6. **Supplementary Fig. S6.** | The salt layer samples collected from Qi Jiao Jing Lake located at Xinjiang province, China.

**Additional file 3.** Description of novel members of *Candidatus* Nanohaloarchaeota.

**Additional file 4.** All commands, scripts, and R codes are included.

## Data Availability

The raw reads of metagenomic sequencing and 16S rRNA-based amplicon sequencing are available in GenBank under BioProject ID PRJNA820349. The three *Ca*. Nanohaloarchaeota MAGs are also publicly available under this BioProject with the following accession numbers: QJJ-5_bin.20 (JALIDO000000000), QJJ-7_bin.66 (JALIDP000000000), and QJJ-9_bin.46 (JALIDQ000000000). A full record of commands and statistical analysis is included in Additional file 4.

## Abbreviations

QJJ: Qi Jiao Jing
MAGs: Metagenome-assembled genomes
ASVs: Amplicon sequence variants
*rbcL*: Ribulose 1,5-bisphosphate carboxylase
pI: Isoelectric points

## References

[1] Offre P, Spang A, Schleper C (2013). Archaea in biogeochemical cycles. Annu Rev Microbiol.

[2] Spang A, Saw JH, Jorgensen SL, Zaremba-Niedzwiedzka K, Martijn J, Lind AE (2015). Complex archaea that bridge the gap between prokaryotes and eukaryotes. Nature.

[3] Lewis WH, Tahon G, Geesink P, Sousa DZ, Ettema TJG (2021). Innovations to culturing the uncultured microbial majority. Nat Rev Microbiol.

[4] Spang A, Caceres EF, Ettema TJG (2017). Genomic exploration of the diversity, ecology, and evolution of the archaeal domain of life. Science.

[5] Baker BJ, De Anda V, Seitz KW, Dombrowski N, Santoro AE, Lloyd KG (2020). Diversity, ecology and evolution of archaea. Nat Microbiol.

[6] Zaremba-Niedzwiedzka K, Caceres EF, Saw JH, Backstrom D, Juzokaite L, Vancaester E (2017). Asgard archaea illuminate the origin of eukaryotic cellular complexity. Nature.

[7] Rinke C, Schwientek P, Sczyrba A, Ivanova NN, Anderson IJ, Cheng JF (2013). Insights into the phylogeny and coding potential of microbial dark matter. Nature.

[8] Rinke C, Chuvochina M, Mussig AJ, Chaumeil PA, Davin AA, Waite DW (2021). A standardized archaeal taxonomy for the Genome Taxonomy Database. Nat Microbiol.

[9] Castelle CJ, Brown CT, Anantharaman K, Probst AJ, Huang RH, Banfield JF (2018). Biosynthetic capacity, metabolic variety and unusual biology in the CPR and DPANN radiations. Nat Rev Microbiol.

[10] Dombrowski N, Lee JH, Williams TA, Offre P, Spang A (2019). Genomic diversity, lifestyles and evolutionary origins of DPANN archaea. FEMS Microbiol Lett.

[11] Beam JP, Becraft ED, Brown JM, Schulz F, Jarett JK, Bezuidt O (2020). Ancestral absence of electron transport chains in Patescibacteria and DPANN. Front Microbiol.

[12] Narasingarao P, Podell S, Ugalde JA, Brochier-Armanet C, Emerson JB, Brocks JJ (2012). De novo metagenomic assembly reveals abundant novel major lineage of archaea in hypersaline microbial communities. ISME J.

[13] Hamm JN, Erdmann S, Eloe-Fadrosh EA, Angeloni A, Zhong L, Brownlee C (2019). Unexpected host dependency of antarctic Nanohaloarchaeota. Proc Natl Acad Sci USA.

[14] La Cono V, Messina E, Rohde M, Arcadi E, Ciordia S, Crisafi F (2020). Symbiosis between nanohaloarchaeon and haloarchaeon is based on utilization of different polysaccharides. Proc Natl Acad Sci USA.

[15] Ghai R, Pasic L, Fernandez AB, Martin-Cuadrado AB, Mizuno CM, McMahon KD (2011). New abundant microbial groups in aquatic hypersaline environments. Sci Rep.

[16] Andrade K, Logemann J, Heidelberg KB, Emerson JB, Comolli LR, Hug LA (2015). Metagenomic and lipid analyses reveal a diel cycle in a hypersaline microbial ecosystem. ISME J.

[17] Vavourakis CD, Ghai R, Rodriguez-Valera F, Sorokin DY, Tringe SG, Hugenholtz P (2016). Metagenomic insights into the uncultured diversity and physiology of microbes in four hypersaline soda lake brines. Front Microbiol.

[18] Crits-Christoph A, Gelsinger DR, Ma B, Wierzchos J, Ravel J, Davila A (2016). Functional interactions of archaea, bacteria and viruses in a hypersaline endolithic community. Environ Microbiol.

[19] Zhao D, Zhang S, Xue Q, Chen J, Zhou J, Cheng F (2020). Abundant taxa and favorable pathways in the microbiome of soda-saline lakes in inner mongolia. Front Microbiol.

[20] Castelle CJ, Méheust R, Jaffe AL, Seitz K, Gong X, Baker BJ (2021). Protein family content uncovers lineage relationships and bacterial pathway maintenance mechanisms in DPANN archaea. Front Microbiol.

[21] Xiang Hui-Ping GT-W, Zhao Shun-Xian, Zhang Xi-Chao, Ou Meng-Ying, Lin Yi-Jin, Wang Peng-Hao. Actinobacterial community and ionic composition in sediment of Xinjiang saline lakes: Barkol, Qijiaojing and Taitema. Microbiol China. 2018; 45:1228–1236.

[22] Vavourakis CD, Andrei A-S, Mehrshad M, Ghai R, Sorokin DY, Muyzer G (2018). A metagenomics roadmap to the uncultured genome diversity in hypersaline soda lake sediments. Microbiome.

[23] Aono R, Sato T, Imanaka T, Atomi H (2015). A pentose bisphosphate pathway for nucleoside degradation in archaea. Nat Chem Biol.

[24] Jaffe AL, Castelle CJ, Dupont CL, Banfield JF, Falush D (2019). Lateral gene transfer shapes the distribution of RuBisCO among candidate phyla radiation bacteria and DPANN archaea. Mol Biol Evol.

[25] Sato T, Atomi H, Imanaka T (2007). Archaeal type III RuBisCOs function in a pathway for AMP metabolism. Science.

[26] Dombrowski N, Williams TA, Sun JR, Woodcroft BJ, Lee JH, Minh BQ (2020). Undinarchaeota illuminate DPANN phylogeny and the impact of gene transfer on archaeal evolution. Nat Commun.

[27] Sorokin DY, Kublanov IV, Gavrilov SN, Rojo D, Roman P, Golyshin PN (2016). Elemental sulfur and acetate can support life of a novel strictly anaerobic haloarchaeon. ISME J.

[28] Sorokin DY, Messina E, Smedile F, Roman P, Damste JSS, Ciordia S (2017). Discovery of anaerobic lithoheterotrophic haloarchaea, ubiquitous in hypersaline habitats. ISME J.

[29] Kamanda Ngugi D, Blom J, Alam I, Rashid M, Ba-Alawi W, Zhang G (2015). Comparative genomics reveals adaptations of a halotolerant thaumarchaeon in the interfaces of brine pools in the Red Sea. ISME J.

[30] Jarrell KF, Ding Y, Nair DB, Siu S (2013). Surface appendages of archaea: structure, function, genetics and assembly. Life.

[31] Comolli LR, Banfield JF (2014). Inter-species interconnections in acid mine drainage microbial communities. Front Microbiol.

[32] Py B, Loiseau L, Barras F (2001). An inner membrane platform in the type II secretion machinery of gram-negative bacteria. Embo Rep.

[33] Szabo Z, Stahl AO, Albers SV, Kissinger JC, Driessen AJ, Pohlschroder M (2007). Identification of diverse archaeal proteins with class III signal peptides cleaved by distinct archaeal prepilin peptidases. J Bacteriol.

[34] Makarova KS, Koonin EV, Albers SV (2016). Diversity and evolution of type IV pili systems in archaea. Front Microbiol.

[35] Lee CJD, McMullan PE, O'Kane CJ, Stevenson A, Santos IC, Roy C (2018). NaCl-saturated brines are thermodynamically moderate, rather than extreme, microbial habitats. FEMS Microbiol Rev.

[36] Andrei AS, Banciu HL, Oren A (2012). Living with salt: metabolic and phylogenetic diversity of archaea inhabiting saline ecosystems. FEMS Microbiol Lett.

[37] Brininger C, Spradlin S, Cobani L, Evilia C (2018). The more adaptive to change, the more likely you are to survive: protein adaptation in extremophiles. Semin Cell Dev Biol.

[38] Karan R, Capes MD, Dassarma S (2012). Function and biotechnology of extremophilic enzymes in low water activity. Aquat Biosyst.

[39] Mevarech M, Frolow F, Gloss LM (2000). Halophilic enzymes: proteins with a grain of salt. Biophys Chem.

[40] Gunde-Cimerman N, Plemenitas A, Oren A (2018). Strategies of adaptation of microorganisms of the three domains of life to high salt concentrations. FEMS Microbiol Rev.

[41] Zhou XX, Wang YB, Pan YJ, Li WF (2008). Differences in amino acids composition and coupling patterns between mesophilic and thermophilic proteins. Amino Acids.

[42] Botting CH, Talbot P, Paytubi S, White MF (2010). Extensive lysine methylation in hyperthermophilic crenarchaea: potential implications for protein stability and recombinant enzymes. Archaea.

[43] Aono R, Sato T, Yano A, Yoshida S, Nishitani Y, Miki K (2012). Enzymatic characterization of AMP phosphorylase and ribose-1,5-bisphosphate isomerase functioning in an archaeal AMP metabolic pathway. J Bacteriol.

[44] Shipman K (2015). Clinical biochemistry: metabolic and clinical aspects. 3rd ed. Ann Clin Biochem..

[45] Jiao J-Y, Fu L, Hua Z-S, Liu L, Salam N (2021). Insight into the function and evolution of Wood-Ljungdahl pathway in Actinobacteria. ISME J.

[46] Caporaso JG, Lauber CL, Walters WA, Berg-Lyons D, Huntley J, Fierer N (2012). Ultra-high-throughput microbial community analysis on the Illumina HiSeq and MiSeq platforms. ISME J.

[47] Walters W, Hyde ER, Berg-Lyons D, Ackermann G, Humphrey G, Parada A (2016). Improved bacterial 16S rRNA gene (V4 and V4–5) and fungal internal transcribed spacer marker gene primers for microbial community surveys. Msystems.

[48] Martin M (2011). Cutadapt removes adapter sequences from high-throughput sequencing reads. EMBnet J.

[49] Bolyen E, Rideout JR, Dillon MR, Bokulich NA, Abnet CC, Al-Ghalith GA (2019). Reproducible, interactive, scalable and extensible microbiome data science using QIIME 2. Nat Biotechnol.

[50] Quast C, Pruesse E, Yilmaz P, Gerken J, Schweer T, Yarza P (2012). The SILVA ribosomal RNA gene database project: improved data processing and web-based tools. Nucleic Acids Res.

[51] Bankevich A, Nurk S, Antipov D, Gurevich AA, Dvorkin M, Kulikov AS (2012). SPAdes: a new genome assembly algorithm and its applications to single-cell sequencing. J Comput Biol.

[52] Kang DD, Li F, Kirton E, Thomas A, Egan R, An H (2019). MetaBAT 2: an adaptive binning algorithm for robust and efficient genome reconstruction from metagenome assemblies. PeerJ.

[53] Chaumeil PA, Mussig AJ, Hugenholtz P, Parks DH (2020). GTDB-Tk: a toolkit to classify genomes with the Genome Taxonomy Database. Bioinformatics..

[54] Parks DH, Chuvochina M, Chaumeil PA, Rinke C, Mussig AJ, Hugenholtz P (2020). A complete domain-to-species taxonomy for bacteria and archaea. Nat Biotechnol.

[55] He C, Keren R, Whittaker ML, Farag IF, Doudna JA, Cate JHD (2021). Genome-resolved metagenomics reveals site-specific diversity of episymbiotic CPR bacteria and DPANN archaea in groundwater ecosystems. Nat Microbiol.

[56] Parks DH, Imelfort M, Skennerton CT, Hugenholtz P, Tyson GW (2015). CheckM: assessing the quality of microbial genomes recovered from isolates, single cells, and metagenomes. Genome Res.

[57] Lagesen K, Hallin P, Rodland EA, Staerfeldt HH, Rognes T, Ussery DW (2007). RNAmmer: consistent and rapid annotation of ribosomal RNA genes. Nucleic Acids Res.

[58] Schattner P, Brooks AN, Lowe TM (2005). The tRNAscan-SE, snoscan and snoGPS web servers for the detection of tRNAs and snoRNAs. Nucleic Acids Res.

[59] Hyatt D, Chen GL, Locascio PF, Land ML, Larimer FW, Hauser LJ (2010). Prodigal: prokaryotic gene recognition and translation initiation site identification. BMC Bioinform.

[60] Buchfink B, Xie C, Huson DH (2015). Fast and sensitive protein alignment using DIANOND. Nat Methods.

[61] Huang L, Zhang H, Wu P, Entwistle S, Li X, Yohe T (2018). dbCAN-seq: a database of carbohydrate-active enzyme (CAZyme) sequence and annotation. Nucleic Acids Res.

[62] Rawlings ND (2006). MEROPS: the peptidase database. Nucleic Acids Res.

[63] Petersen TN, Brunak S, von Heijne G, Nielsen H (2011). SignalP 4.0: discriminating signal peptides from transmembrane regions. Nat Methods.

[64] Kozlowski LP (2016). IPC - Isoelectric Point Calculator. Biol Direct.

[65] Hug LA, Baker BJ, Anantharaman K, Brown CT, Probst AJ, Castelle CJ (2016). A new view of the tree of life. Nat Microbiol.

[66] Wu M, Scott AJ (2012). Phylogenomic analysis of bacterial and archaeal sequences with AMPHORA2. Bioinformatics.

[67] Edgar RC (2004). MUSCLE: multiple sequence alignment with high accuracy and high throughput. Nucleic Acids Res.

[68] Capella-Gutierrez S, Silla-Martinez JM, Gabaldon T (2009). TrimAl: a tool for automated alignment trimming in large-scale phylogenetic analyses. Bioinformatics.

[69] Fu LM, Niu BF, Zhu ZW, Wu ST, Li WZ (2012). CD-HIT: accelerated for clustering the next-generation sequencing data. Bioinformatics.

[70] Nguyen LT, Schmidt HA, von Haeseler A, Minh BQ (2015). IQ-TREE: a fast and effective stochastic algorithm for estimating maximum-likelihood phylogenies. Mol Biol Evol.

[71] Letunic I, Bork P (2007). Interactive Tree of Life (iTOL): an online tool for phylogenetic tree display and annotation. Bioinformatics.

[72] Enright AJ, Van Dongen S, Ouzounis CA (2002). An efficient algorithm for large-scale detection of protein families. Nucleic Acids Res.

[73] Szollosi GJ, Rosikiewicz W, Boussau B, Tannier E, Daubin V (2013). Efficient exploration of the space of reconciled gene trees. Syst Biol.

[74] Szollosi GJ, Davin AA, Tannier E, Daubin V, Boussau B (2015). Genome-scale phylogenetic analysis finds extensive gene transfer among fungi. Philos T R Soc B.

[75] Martijn J, Schon ME, Lind AE, Vosseberg J, Williams TA, Spang A (2020). Hikarchaeia demonstrate an intermediate stage in the methanogen-to-halophile transition. Nat Commun.

