# Supplementary material for: Functional differentiation determines the molecular basis of the symbiotic lifestyle of Ca. Nanohaloarchaeota
Source: Microbiome. 2022 Oct 14;10:172. doi: 10.1186/s40168-022-01376-y (PMC9563170; doi:10.1186/s40168-022-01376-y)
Supplement: Supplementary file 3 — Additional file 2: Supplementary Fig. S1-S6. Supplementary Fig. S1. | Phylogenetic placement of Ca. Nanohaloarchaeota MAGs based on 122 concatenated archaeal protein markers. Supplementary Fig. S2. | Pairwise comparisons of average amino acid identities among all Ca. Nanohaloarchaeota genomes. Supplementary Fig. S3. | The gene clusters related to the pili biosynthesis. Supplementary Fig. S4. | Maximum likelihood-based phylogenetic tree of alpha amylase encoded by amy using IQ-TREE with the best model of LG+R5. Supplementary Fig. S5. | Maximum likelihood-based phylogenetic tree of glycogen debranching enzyme encoded by AGL using IQ-TREE with the best model of LG+F+R6. Supplementary Fig. S6. | The salt layer samples collected from Qi Jiao Jing Lake located at Xinjiang province, China. [file 40168_2022_1376_MOESM2_ESM.docx]

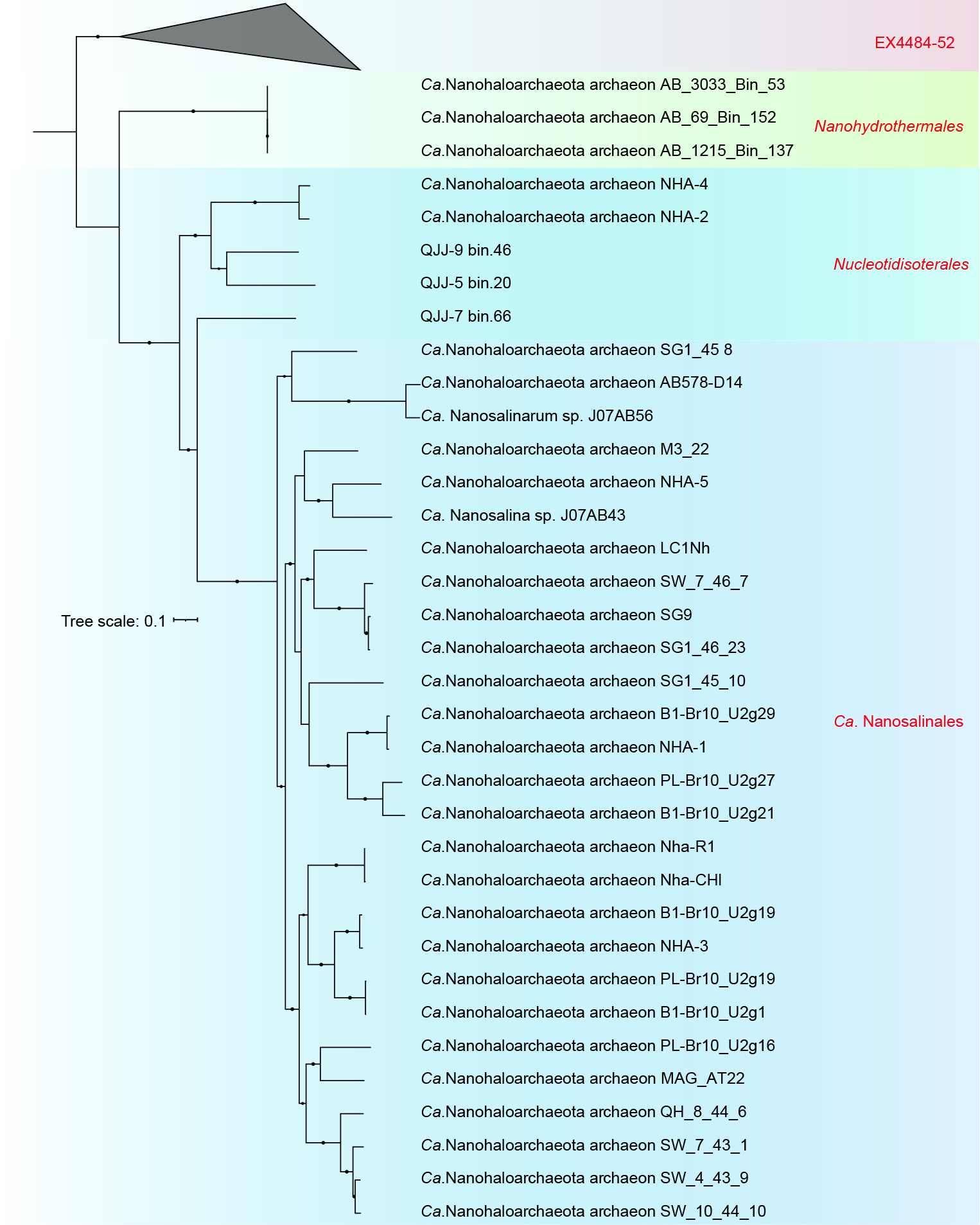


Supplementary Fig. S1 | Phylogenetic placement of MAGs based on 122 concatenated archaeal protein markers. The phylogeny was constructed using IQ-TREE with the best model of LG+F+R4. Bootstrap values were calculated based on 1000 replicates and nodes with percentages > 70% are indicated as black circles.


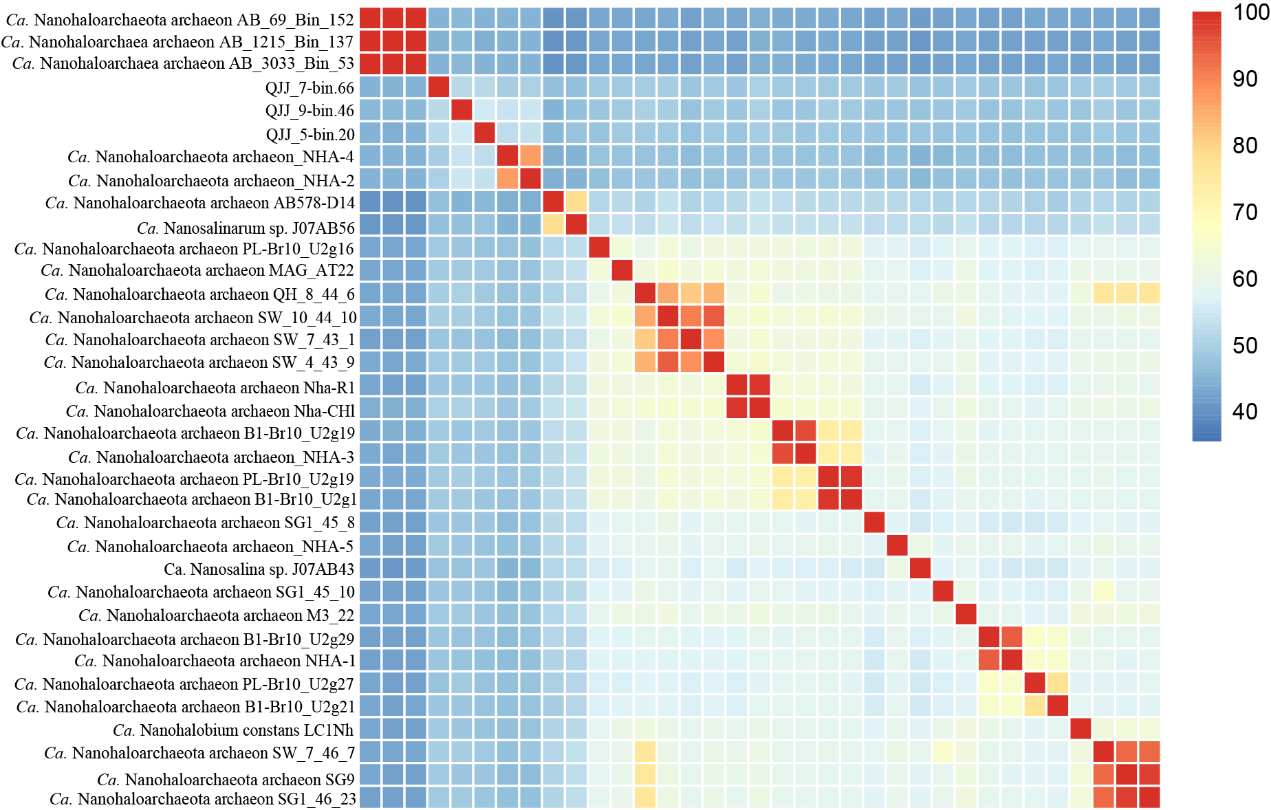
Supplementary Fig. S2 | Pairwise comparisons of average amino acid identities among all *Ca.* Nanohaloarchaeota genomes.


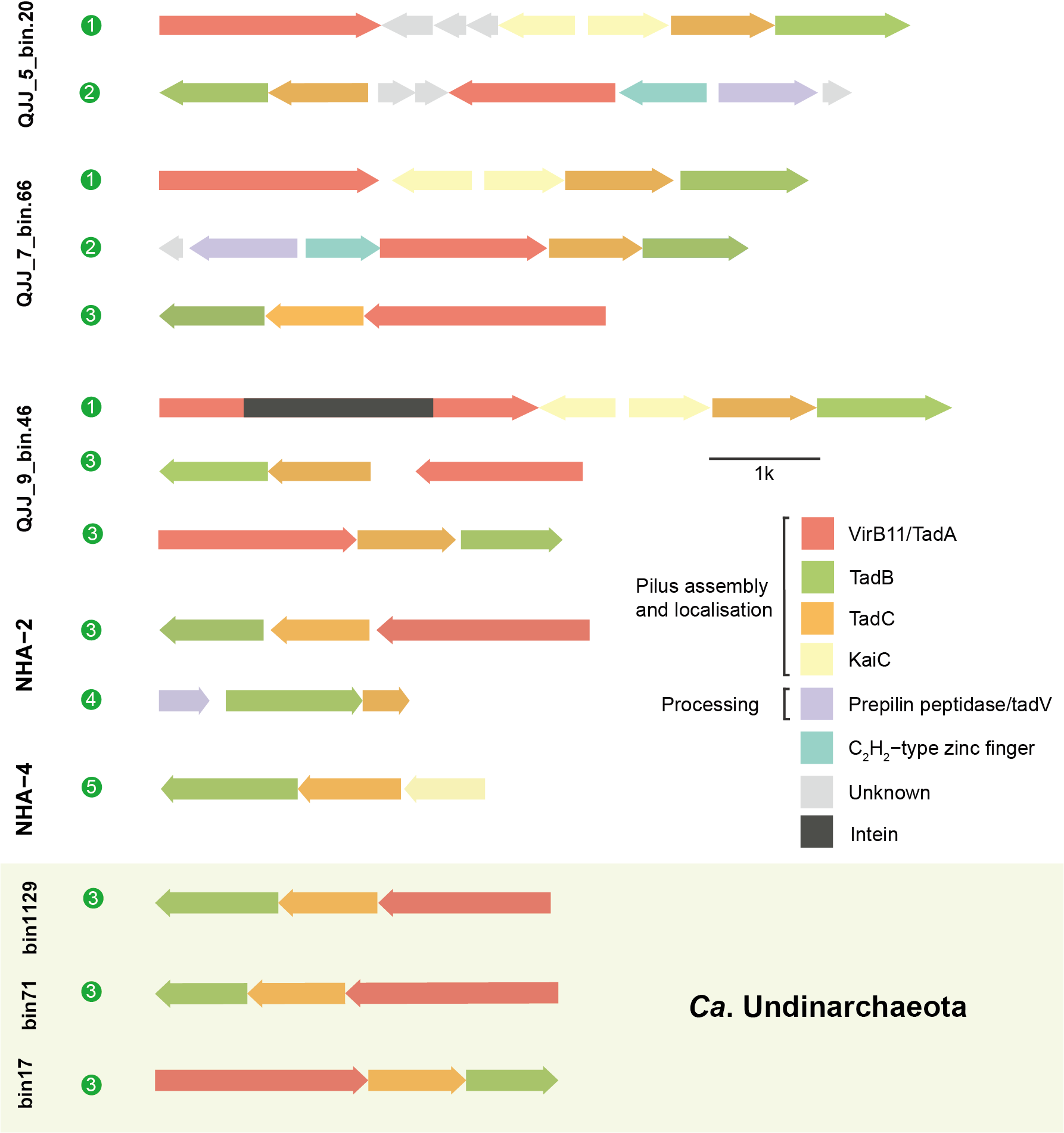


**Supplementary Fig. S3 | The gene clusters related to the pili biosynthesis.**


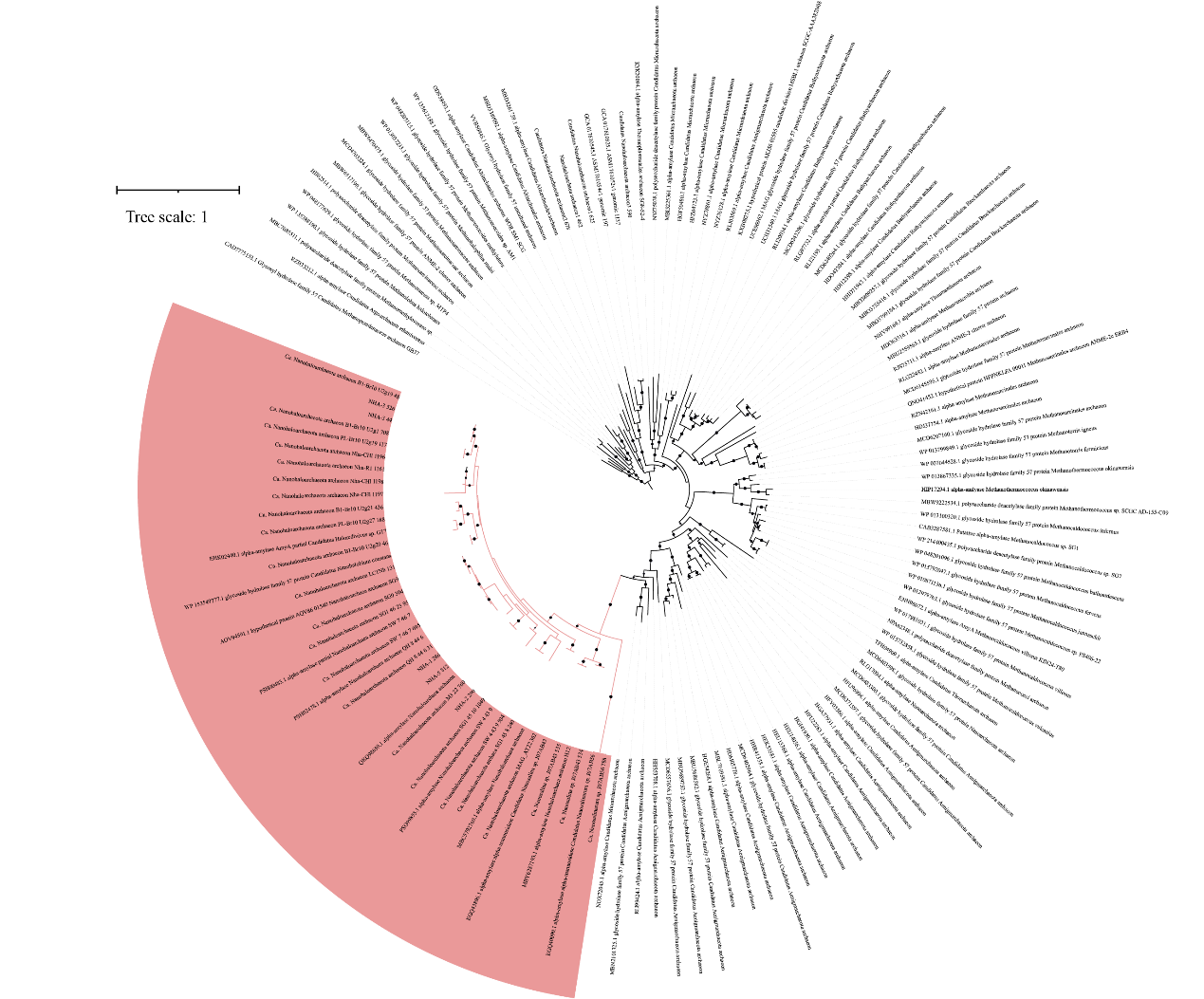


Supplementary Fig. S4 | Maximum likelihood-based phylogenetic tree of alpha amylase encoded by amy using IQ-TREE with the best model of LG+R5. Bootstrap values were calculated based on 1000 replicates and nodes with percentages > 70% are indicated as black circles. Items in red represent *amy* genes identified from *Ca.* Nanohaloarchaeota.


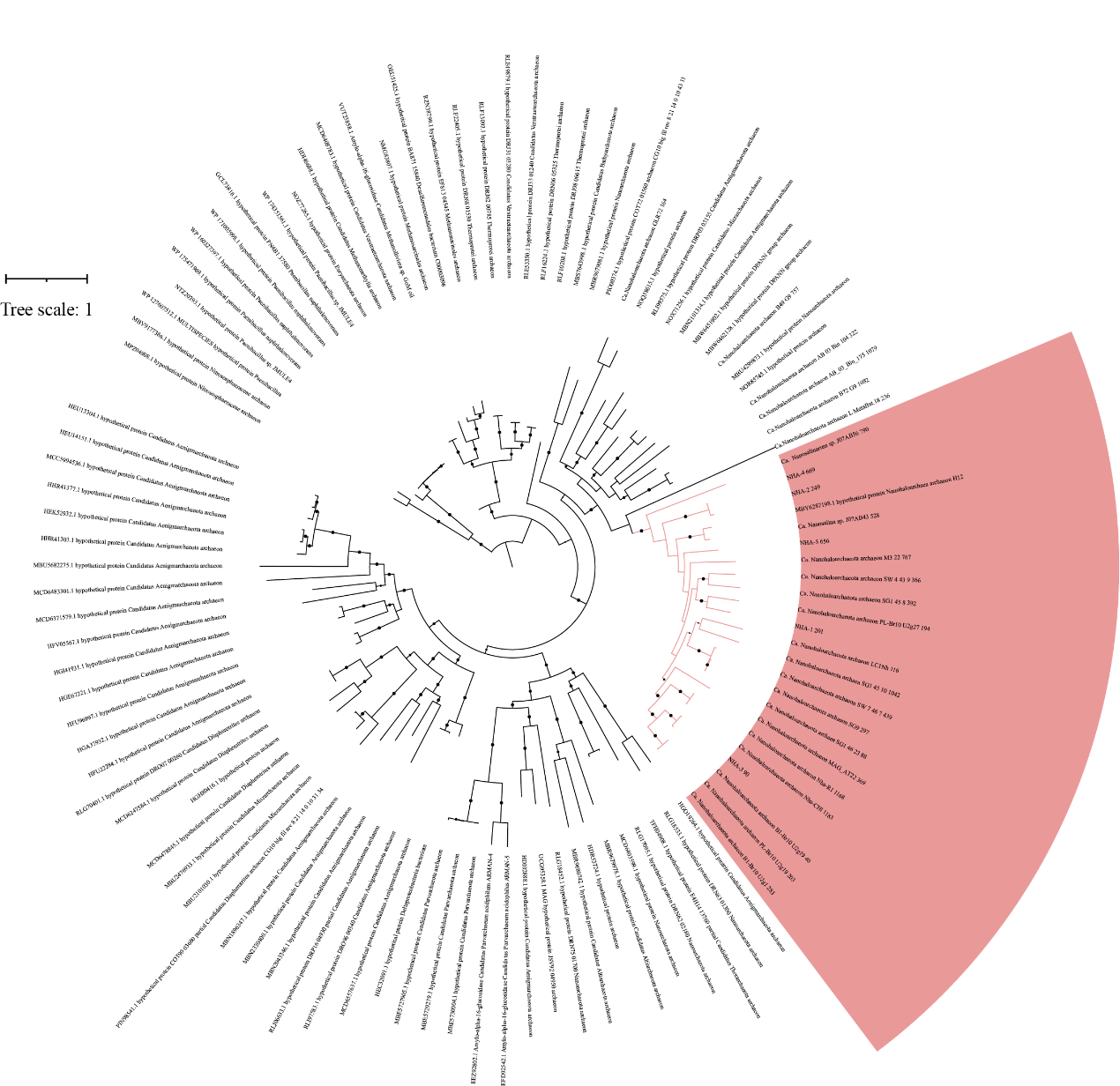


**Supplementary Fig. S5 | Maximum likelihood-based phylogenetic tree of glycogen debranching enzyme** encoded by AGL **using IQ-TREE with the best model of LG+F+R6.** Bootstrap values were calculated based on 1000 replicates and nodes with percentages > 70% are indicated as black circles. Items in red represent AGL genes identified from *Ca.* Nanohaloarchaeota.


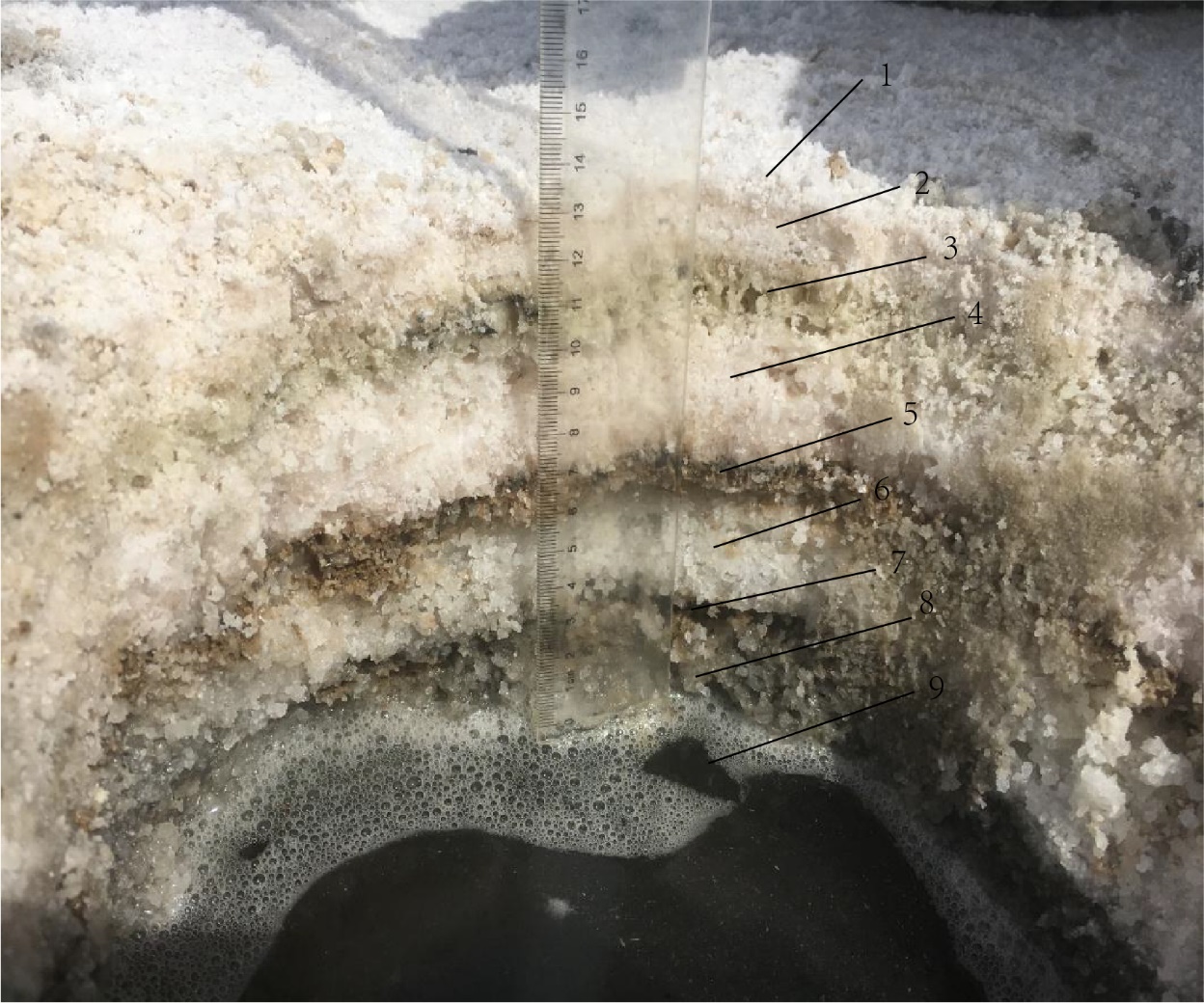


Supplementary Fig. S6 | The salt layer samples collected from Qi Jiao Jing Lake located at Xinjiang province, China.
