# Supplementary material for: Functional differentiation determines the molecular basis of the symbiotic lifestyle of Ca. Nanohaloarchaeota
Source: Microbiome. 2022 Oct 14;10:172. doi: 10.1186/s40168-022-01376-y (PMC9563170; doi:10.1186/s40168-022-01376-y)
Supplement: Supplementary file 4 — Additional file 3. Description of novel members of Candidatus Nanohaloarchaeota. [file 40168_2022_1376_MOESM3_ESM.docx]

**Description of novel members of *Candidatus* Nanohaloarchaeota**

**Description of** ***Nucleotidisoterales* ord. nov.**

Nu.cle.o.to.di.so.ter.a’les (N.L. masc. n. *Nucleotidisoter*, rescuer of nucleotides, referring to the nucleotide salvage pathways predicted in these organisms; L. fem. pl. suff. *-ales*, ending to denote an order; N.L. fem. pl. n. *Nucleotidisoterales*, the order whose nomenclatural type is the genus *Nucleotidisoter*)

This order belongs to the class *Ca.* Nanosalinia, in the phylum *Ca.* Nanohaloarchaeota. Based on phylogenomic analyses and relative evolutionary divergence (RED), this lineage represents a monophyletic order-level group. Genomes belonging to this order encode core cellular functional genes involved in DNA replication, transcription, and translation, but lack genes involved in nucleotide, amino acid, lipid, and cofactor biosynthesis, indicative of potential symbiotic dependencies. Complete nucleotide salvage pathways are conserved within the known genomes belonging to this order and is phylogenetically related to genes from other DPANN archaea. A lack of genes coding for an electron transport chain indicates that the lineage may be strictly anaerobic. Genes encoding several proteases (mainly different peptidases), and nucleases make up a repertoire of genes potentially involved in scavenging and recycling of extracellular DNA and proteins. Genes for archaellum biosynthesis are lacking from all known genomes belonging to this order, however multiple putative pili biosynthesis and assembly clusters are found in the genomes. The enrichment for the use of highly acidic and hydrophilic amino acids in protein-coding genes, as well as low median isoelectric points for these predicted proteins, likely contribute to survival in high salt environments. All known genomes belonging to this order has been recovered from hypersaline metagenomic samples. The nomenclatural type for the order is the genus *Nucleotidisoterales*.

**Description of *Candidatus* Nucleotidisoteraceae fam. nov.**

Nu.cle.o.to.di.so.ter.a’ce.ae (N.L. masc. n. *Nucleotidisoter*, rescuer of nucleotides, referring to the nucleotide salvage pathways predicted in these organisms; L. fem. pl. suff. *-aceae*, ending to denote a family; N.L. fem. pl. n. *Nucleotidisoteraceae*, family of the genus *Candidatus* Nucleotidisoter)

This family belongs to the order *Nucleotidisoterales*, in the class *Ca.* Nanosalinia, in the phylum *Ca.* Nanohaloarchaeota. RED and Average Amino acid Identity (AAI) support the novelty of this taxon as a family. As a monotypic taxon, the description of this family is currently the same as for the genus *Candidatus* Nucleotidisoter, the nomenclatural type for the family.

**Description of *Candidatus* Nucleotidisoter** **gen. nov.**

Nu.cle.o.to.di.so’ter (N.L. neut. n. *nucleotidum*, nucleotide or nucleoside; N.L. masc. n. soter, rescuer; N.L. masc. n. *Nucleotidisoter*, rescuer of nucleotides, referring to the nucleotide salvage pathways predicted in these organisms)

This genus belongs to the family *Candidatus* Nucleotidisoteraceae, in the order *Nucleotidisoterales*, in the class *Ca.* Nanosalinia, phylum *Ca.* Nanohaloarchaeota. Only one genome belonging to this genus is available currently, and the description of this genus is the same as for the species *Candidatus* Nucleotidisoter xinjiangensis, the nomenclatural type for the genus.

**Description of *Candidatus* Nucleotidisoter xinjiangensis sp. nov.**

xin.jiang.en’sis (N.L. masc. adj. *xinjiangensis*, of Xinjiang, referring to the Xinjiang Uygur autonomous region of China where the genome of this organism was recovered from)

This species is the nomenclatural type for the genus *Candidatus* Nucleotidisoter. The genome of this organism was recovered from metagenomic sequencing efforts of salt layer samples, taken from QiJiaoJing Lake, Xinjiang, China. The available genome is estimated based on marker gene presence as 87.5 % complete, with 0.07 % contamination. High strain heterogeneity is observed in the available metagenome-assembled genome, indicating a genetically diverse population for this organism. The genome has an estimated genome size of 701,335 bp and a GC content of 50.48 % in 48 scaffolds. The genes for complete nucleotides salvage pathway were predicted from this genome. The nomenclatural type for this species is the genome QJJ-5_bin.20 (JALIDO000000000), recovered from a hypersaline lake in China.

**Description of *Nucleotidivindicaceae* fam. nov.**

Nu.cle.o.to.di.vin.dic.a’ce.ae (N.L. masc. n. *Nucleotidivindex*, rescuer of nucleotides, referring to the nucleotide salvage pathways predicted in these organisms; L. fem. pl. suff. *-aceae*, ending to denote a family; N.L. fem. pl. n. *Nucleotidivindicaceae*, family of the genus *Nucleotidivindex*)

This family belongs to the order *Nucleotidisoterales*, in the class *Ca.* Nanosalinia, in the phylum *Ca.* Nanohaloarchaeota. The novelty of this family is supported by AAI and RED. As a monotypic taxon, the description of this family is currently the same as for the genus *Nucleotidivindex*, the nomenclatural type for the family.

**Description of *Nucleotidivindex* gen. nov.**

Nu.cle.o.to.di.vin’dex (N.L. neut. n. nucleotidum, nucleotide or nucleoside; N.L. masc. n. vindex, rescuer, vindicator; N.L. masc. n. *Nucleotidivindex*, rescuer of nucleotides, referring to the nucleotide salvage pathways predicted in these organisms)

This genus belongs to the family *Nucleotidivindexaceae*, in the order *Nucleotidisoterales*, in the class *Ca.* Nanosalinia, phylum *Ca.* Nanohaloarchaeota. Only one genome belonging to this genus is available currently, and the description of this genus is the same as for the species *Nucleotidivindex* *qijiaojingensis* the nomenclatural type for the genus.

**Description of *Nucleotidivindex*** ***qijiaojingensis* sp. nov.**

qi.jiao.jing.en’sis (N.L. masc. adj. *qijiaojingensis*, of QiJiaoJing Lake, in Xinjiang province in China where the genome of this organism was recovered from)

This species is the nomenclatural type for the genus *Nucleotidivindex*. The genome of this organism was recovered from metagenomic sequencing efforts of salt layer samples, taken from QiJiaoJing Lake, Xinjiang, China. The estimated genome completeness based on marker gene presence is 95.8 % complete, with 0.93 % contamination, and high strain heterogeneity, indicative of high genetic diversity within this population. The estimated genome size is 753,270 bp and the GC content of the genome is 52.42 % in 34 scaffolds. The genes for complete nucleotides salvage pathway were predicted from this genome. The nomenclatural type for this species is the genome QJJ-7_bin.66 (JALIDP000000000), recovered from a hypersaline lake in China.

**Description of *Candidatus* Nanosalenecusaceae fam. nov.**

Na.no.sal.e.ne.cus.a’ce.ae (N.L. masc. n. *Nanosalenecus*, a small inhabitant of salt/brine; L. fem. pl. suff. *-aceae*, ending to denote a family; N.L. fem. pl. n. *Nanosalenecusaceae*, family of the genus *Candidatus* Nanosalenecus)

This family belongs to the order *Nucleotidisoterales*, in the class *Ca.* Nanosalinia, in the phylum *Ca.* Nanohaloarchaeota. Designation as a novel family of this taxon is supported by AAI and RED. As a monotypic taxon, the description of this family is currently the same as for the genus *Candidatus* Nanosalenecus, the nomenclatural type for the family.

**Description of *Candidatus* *Nanosalenecus* gen. nov.**

Na.no.sal.e.ne’cus (Gr. masc. n. nânos, a dwarf; L. masc. n. sal, salt or brine; N.L. masc. n. enecus, an inhabitant; N.L. masc. n. *Nanosalenecus*, a small inhabitant of salt/brine)

This genus belongs to the family *Candidatus* Nanosalenecusaceae, in the order *Nucleotidisoterales*, in the class *Ca.* Nanosalinia, phylum *Ca.* Nanohaloarchaeota. Only one genome belonging to this genus is available currently, and the description of this genus is the same as for the species *Candidatus* Nanosalenecus halilacustris the nomenclatural type for the genus.

**Description of *Candidatus*** **Nanosalenecus** **halilacustris sp. nov.**

ha.li.la.cus’tris (Gr. fem. n. hals, salt; N.L. masc. n. lacustris, of a lake; N.L. masc. adj. *halilacustris*, of a saline lake)

This species is the nomenclatural type for the genus *Candidatus* Nanosalenecus. The genome of this organism was recovered from metagenomic sequencing efforts of saline water samples, taken from QiJiaoJing Lake, Xinjiang, China. The available genome is estimated as 89.6 % complete, with 0.0 % contamination. The genome is estimated as 625,082 bp, with a GC content of 43.85 % in 34 scaffolds. Nearly complete nucleotides salvage pathway and 6 glycosyltransferases of family 2 (GT2) and 4 (GT4) genes were predicted from this genome. The nomenclatural type for this species is the genome QJJ-9_bin.46 (JALIDQ000000000), recovered from a hypersaline lake in China.

**Description of *Nanohydrothermales* ord. nov.**

Na.no.hy.dro.ther.ma’les (N.L. masc. n. *Nanohydrothermus*, small organism from hot water; L. fem. pl. suff. *-ales*, ending to denote an order; N.L. fem. pl. n. *Nanohydrothermales*, the order whose nomenclatural type is the genus *Nanohydrothermus*)

This order belongs to the class *Ca.* Nanosalinia, in the phylum *Ca.* Nanohaloarchaeota. Phylogenomic analyses and RED support delineation of this taxon as a novel order. As a monotypic order, the description is currently the same as for the type genus, Nanohydrothermus.

**Description of *Nanohydrothermaceae* fam. nov.**

Na.no.hy.dro.ther.ma’ce.ae (N.L. masc. n. *Nanohydrothermus*, small organism from hot water; L. fem. pl. suff. *-aceae*, ending to denote a family; N.L. fem. pl. n. *Nanohydrothermaceae*, family of the genus *Nanohydrothermus*)

This family belongs to the order *Nanohydrothermales*, class *Ca.* Nanosalinia, in the phylum *Ca.* Nanohaloarchaeota. As a monotypic order, the description is currently the same as for Nanohydrothermus, the nomenclatural type for the family.

**Description of *Nanohydrothermus* gen. nov.**

Na.no.hy.dro.ther’mus (Gr. masc. n. *nânos*, a dwarf; Gr. neut. n. *hydor*, water; Gr. masc. adj. *thermos*, hot; N.L. masc. n. *Nanohydrothermus*, small organism from hot water)

This genus belongs to the family *Nanohydrothermaceae*, in the order *Nanohydrothermales*, class *Ca.* Nanosalinia and the phylum *Ca.* Nanohaloarchaeota. As a monotypic genus, the description for this genus is currently the same as for the species *Nanohydrothermus* guaymasensis, the nomenclatural type for the genus.

**Description of *Nanohydrothermus* *guaymasensis* sp. nov.**

guay.ma.sen’sis (N.L. masc. adj. *guaymasensis*, of Guaymas, referring to the Guaymas Basin in the Gulf of California, where the genome of this organism was recovered from)

This species is the type for the genus *Nanohydrothermus*. Three genomes representing this species is available, with pairwise AAI values above 99 %, and all genomes of this organism were recovered from sediment samples between 6 and 33 cm deep, at a depth of 2,000 m in the Gauymas Basin, in the Gulf of California. This species represents the first thermophilic *Ca.* Nanohaloarchaeota recovered. Estimated genomes ranged between 640,728 and 788,277 bp, in 10 to 28 scaffolds. Estimated completeness ranged between 87.5% and 93.75%, with very low to no contamination. The GC content of genomes were between 32.17 and 32.36 %. Compared to other *Ca.* Nanohaloarchaeota, predicted proteomes for this organism consists of proteins with predicted higher isoelectric points and amino acid usage more similar to other thermophilic DPANN taxa, which can enhance protein stability under higher temperatures. The nomenclatural type for the species is the genome AB_69_Bin_152 (GenBank assembly accession number: GCA_017610085.1).
